# A dataset to assess mobility changes in Chile following local quarantines

**DOI:** 10.1038/s41597-022-01893-3

**Published:** 2023-01-03

**Authors:** Luca Pappalardo, Giuliano Cornacchia, Victor Navarro, Loreto Bravo, Leo Ferres

**Affiliations:** 1 ISTI-CNR, Pisa, Italy; 2 Department of Computer Science, University of Pisa, Pisa, Italy; 3 Faculty of Engineering, Universidad del Desarrollo, Santiago, Chile; 4 Telefónica R&D Santiago, Providencia, Chile; 5 ISI Foundation, Turin, Italy

**Keywords:** Scientific data, Epidemiology

## Abstract

Fighting the COVID-19 pandemic, most countries have implemented non-pharmaceutical interventions like wearing masks, physical distancing, lockdown, and travel restrictions. Because of their economic and logistical effects, tracking mobility changes during quarantines is crucial in assessing their efficacy and predicting the virus spread. Unlike many other heavily affected countries, Chile implemented quarantines at a more localized level, shutting down small administrative zones, rather than the whole country or large regions. Given the non-obvious effects of these localized quarantines, tracking mobility becomes even more critical in Chile. To assess the impact on human mobility of the localized quarantines, we analyze a mobile phone dataset made available by Telefónica Chile, which comprises 31 billion eXtended Detail Records and 5.4 million users covering the period February 26th to September 20th, 2020. From these records, we derive three epidemiologically relevant metrics describing the mobility within and between comunas. The datasets made available may be useful to understand the effect of localized quarantines in containing the COVID-19 pandemic.

## Abstract (summary)

**Table:** 

Measurement(s)	human mobility inter- and intra-administrative country areas
Technology Type(s)	mobile phone records
Sample Characteristic - Organism	Homo sapiens
Sample Characteristic - Environment	city
Sample Characteristic - Location	Chile

## Background & Summary

As of September 2022, the COVID-19 pandemic is a global threat that resulted in around 600 million infected people and more than six million deaths globally^1^. In South America, Chile is among the most severely affected countries, with more than 4.6 million infected people and a death toll that surpassed the 60,000 mark as of September 2022. Similarly to other severely affected countries^2–9^, Chile implemented Non-Pharmaceutical Interventions (NPIs) such as regional lockdown, stay-at-home orders, and travel restrictions, in an attempt to mitigate the COVID-19 epidemics through reducing individual mobility and promoting social distancing. In contrast with countries such as China, Italy, and the USA, which implemented NPIs at the national or regional level^2,6,8,9^, Chile’s implemented NPIs at the comuna level. Comunas, also known as municipalities or communes in other countries, are the smallest administrative (political) subdivision in Chile^10,11^. There are 346 comunas in Chile. Without counting Antártica, the largest comuna with an area of 1.25 million square kilometres, the remaining 345 comunas have a mean area of 2,199 km^2^ (stdev. 4,824 km^2^), with the smallest being San Ramón, Lo Prado, Lo Espejo, and Independencia, with 7 km^2^ each, and the largest is Natales in the Magallanes region, with an area of 4,8974 km^2^. Two comunas in the same region may be regulated by different NPIs: whereas one is in lockdown, adjacent ones might have no travel restrictions. Only one comuna, Santiago, was split in half in terms of NPIs, with one half under quarantine while the other not. Given the peculiarity of NPIs’ spatial scale in Chile, tracking mobility changes at the comuna level is crucial to assess local quarantines’ efficacy and measure the effect of mobility reductions on predicting the virus spread^12^. While indices of changes in human mobility do exist at the regional level in Chile (e.g., the Google Mobility Reports^13^), there are no official indices at the comuna level.

Mobile phone records provide an unprecedented opportunity in tracking human movements^14–18^, allowing for estimating presences and population density^19–21^, mobility patterns^16,22–26^, flows^27–30^, and socio-economic status^31–36^. When used correctly and adequately aggregated to preserve privacy^37–40^, mobile phone data represent a crucial tool for supporting public health actions across the phases of the COVID-19 pandemic^12,41^. Motivated by the potential of mobile phone data in capturing the geographical spread of epidemics^42–45^, researchers and governments have started to collaborate with mobile network operators to estimate the effectiveness of control measures in several countries^2,10,46–50^.

To assess the impact of the NPIs imposed by Chilean authorities in response to the epidemics, we analyse a mobile phone dataset provided by Telefónica Chile, which comprises 31 billion eXtended Detail Records (XDRs) and 5.4 million users distributed all over the country covering the period February 26th, 2020 to September 20th, 2020. An XDR is created every fifteen minutes if a certain threshold of traffic has been reached, thus describing individual movements in great detail^21^. From the XDRs, we derive three epidemiologically relevant metrics: the Index of Internal Mobility (IM_*int*_), which quantifies the amount of mobility within each comuna of the country; the Index of External Mobility (IM_*ext*_), quantifying the mobility between comunas; and the Index of Mobility (IM), which considers any movement, both within and between comunas. We analyse how these metrics change as the COVID-19 epidemics spread out in Chile, highlighting a considerable heterogeneity of response to local quarantines across the country.

The datasets we make available will grow as time goes by and, to the best of our knowledge, are the only ones describing mobility changes and dates of local quarantines in Chile at the comuna level. They can be used not only for fighting against the COVID-19 epidemics but will also benefit other research and applications such as emergency response^51,52^ and crowd flow prediction^14,53–55^. The datasets described are currently used at all levels of the Chilean government.

## Methods

**Fig. 1:**
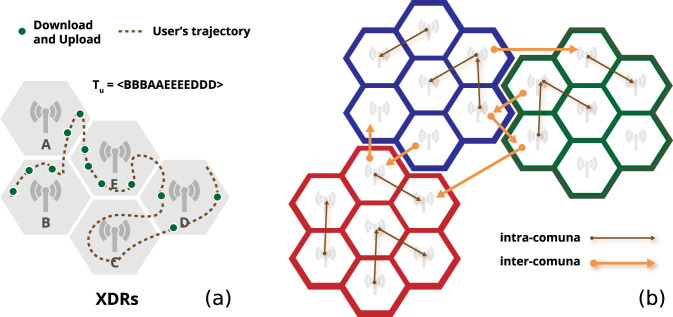
(**a**) Illustrative example of eXtended Detail Records (XDRs) of a mobile phone user. The hexagons represent mobile phone towers and green dots the positions where the user starts a download/upload operation. The dotted line indicates the real movement of the user, from the left to the right. (**b**) Intra-comuna movements (black arrows) and inter-comuna movements (orange arrows). Hexagons of the same color indicate towers that fall in the same comuna.

Mobile phone operators collect several different streams of mobile phones interaction with the cellular network for billing and operational purposes. Among them are the eXtended Detail Records (XDRs), a mixture of human- and device-driven event, triggered either by explicitly requesting an HTTP address or automatically downloading content from the Internet (e.g., emails) every 15 minutes and at certain traffic thresholds. Formally, an XDR is a tuple (*u, t, A, k*), in which there is only one tower *A* involved, *u* is the caller’s identifier, *t* is a timestamp of when the record is created, and *k* is the amount of downloaded information (Fig. 1a). Rather than capturing trips, we are interested in detecting any “movement”, i.e., any transition between two antennas. From an epidemiological point of view, transitions provide a useful indication of people’s displacements and hence useful information about the movements of the virus between areas within the same comuna or between two comunas. Even if an individual’s movement between two antennas may not be a trip from a semantic point of view, it denotes the movement of the virus between those two antennas anyway. To this purpose, from the XDRs of the individuals, we define two types of movement. Every time a user moves from one tower to another within the same comuna, they generate an intra-comuna movement. Every time the user moves from an tower to another in a different comuna, they generate an inter-comuna movement (Fig. 1b). For each day and comuna, we construct three indicators of mobility based on the intra- and inter-comuna movements:

1. *IM*_*int*_ (Index of Internal Mobility), the number of intra-comuna movements for that day;
2. *IM*_*ext*_ (Index of External Mobility), the number of inter-comuna movements for that day;
3. IM = IM_*int*_ + IM_*ext*_ (Index of Mobility).

All the three indices ranges in [0, ∞), where a value of 0 indicates no mobility at all. We normalize the three indices with respect to the number of users that reside in the comuna, estimated as the total number of unique mobile devices whose home tower falls in that comuna. Each device’s home tower is computed as the tower in which it has the highest number of XDRs during nighttime (between 7 pm and 7am, inclusive)0^21,56^. The number of estimated resident users in the comunas is strongly correlated (R^2^ = 0.96, slope = 4.37, intercept = 298.30) with the official population of the comunas as per the official 2017 Chilean Census.

## Data Records

The raw datasets were provided by Telefónica/Movistar Chile, a mobile phone company which possesses between 29–32% of the Chilean mobile phone market. Telefónica gathers data for billing purposes and for network maintenance purposes by persisting network events. Users are not allowed to “opt-out” of billing information, as stated in the terms and conditions below. They are, however, able to opt out of the use of personal data by calling a number or visiting the Telefónica website (see page 3, section 6) of Telefónica’s Terms and Conditions (see^57^ in Spanish). In this study, no personal data or information whatsoever is used in the creation of the dataset proposed here (in fact, it’s only the aggregated number of transitions between rtowe)s, without any individual information.

From the raw datasets we construct the three mobility indices described above. The datasets are released under the CC BY 4.0 License and are publicly available at^58^. Table 1 shows the structure of the dataset describing the mobility indices. Each record refers to a comuna in Chile and describes:

- the official name of the region (region, type:string);
- the identifier of the region as per the official 2017 Chilean Census (rid, type:string);
- the official name of the comuna (comuna, type:string);
- the identifier of the comuna as per the official 2017 Chilean Census (cid, type:string). All maps and their official identifiers can be downloaded from the National Statistics Office of Chile^59^;
- the area of the comuna in km^2^ (area, type:float);
- the values of IM, IM_*int*_ and IM_*ext*_ for that day (type:float);
- the day the IM, IM_*int*_ and IM_*ext*_ values refer to (date, type:date).

**Table 1:** Structure of the released dataset.

region	rid	comuna	cid	area	IM_*int*_	IM_*ext*_	IM	date
Los Ríos	14	Valdivia	14101	1018.32	6.21	0.91	7.13	2020-02-26
Los Ríos	14	Valdivia	14101	1018.32	6.42	0.93	7.35	2020-02-27
Los Ríos	14	Valdivia	14101	1018.32	6.75	1.08	7.84	2020-02-28
Los Ríos	14	Valdivia	14101	1018.32	6.88	1.17	8.05	2020-02-29
Los Ríos	14	Valdivia	14101	1018.32	5.58	1.05	6.63	2020-03-01

Table 2 shows the structure of the quarantines dataset. Each record refers to a quarantine regulation and describes:

- the identifier of the quarantine regulation (qid, type:integer);
- the official name of the comuna (comuna, type:string);
- the status of the quarantine, that can be either active or not active (status, type:string);
- the coverage of the quarantine, that can be either partial, rural, or complete (coverage, type:string);
- the date the quarantine started (start, type:date);
- the date the quarantine ended, which is “ - ” if it is still active (end, type:date);
- the identifier of the comuna as per the official 2017 Chilean Census (cid, type:string);
- the area of the quarantine in m^2^ (area, type:float);
- the perimeter of the quarantine (perimeter, type:float).

**Table 2:** Structure of the quarantines dataset.

qid	comuna	status	coverage	start	end	cid	area	perimeter
4	El Bosque	Active	whole	2020-04-16	—	13105	2.06e7	1.87e4
26	Quinta Normal	Active	whole	2020-04-23	—	13126	1.70e7	2.12e4
38	Cerrillos	Active	whole	2020-05-05	—	13102	2.41e7	2.52e4
42	Conchalí	Active	whole	2020-05-08	—	13104	1.59e7	1.68e4

A limitation of all phone-records studies concerns the position of towers and the geographical area they “illuminate” or serve given their technical specifications. There may be towers that serve two neighboring comunas, for example, impacting our movement counts. However, two phenomena mitigate this problem: *(i)* comunas are generally large, and eventual borderline events are scarce given the 15-minute span; and *(ii)* telco companies do not record all antenna interactions by mobile devices, because storing all that information would be costly. In our case, an event (a phone record or XDR) is typically generated every *y* minutes and if and only if the device has crossed a threshold of *x* MegaBytes (MBs) of traffic (not revealed by the company as it is an industrial secret). A two-rule heuristic determines the quantities *x* and *y*. A “clock” triggers a rule every 15 minutes: if the user has reached *x* MBs at either 15, 30, or 45 minutes, the system appends a new XDR in the database. Some heavy users will use up the *x* MBs threshold at 15 minutes (if they are watching movies on the web, for instance), most at 30 minutes, and a few light users will reach the threshold at 45 minutes. There is also a fair share of frequency at other times. The second rule states that if the control plane of the mobile network notices some particular phone events, such as some antenna handovers, turning off the phone, or losing connection, then a record is created into the database at any time (irrespective of the megabytes used), making it possible to find events anywhere in-between the clock’s 15-minute triggers.

## Technical Validation

In our analysis, we consider two periods: the pre-quarantine period, from March 9th to March 15th, 2020, and the quarantine period, from June 22nd to June 28th, 2020. Although we have two weeks before March 9th, the transition from February to March marks the start of the Fall school semester in Chile. In 2020, March 6th was the start of the semester, so we assume that the “business as usual” period would be best represented by the week of March 9th until March 15th. March 16th marked the start of NPIs in Chile, with the closure of schools, universities and large public gatherings. After that, on March 26th, there was a partial lockdown of seven comunas in the Metropolitan Region. By June 22–28, more than half of the population of the country was under quarantine, and mobility was at 40% reduction.

**Fig. 2:**
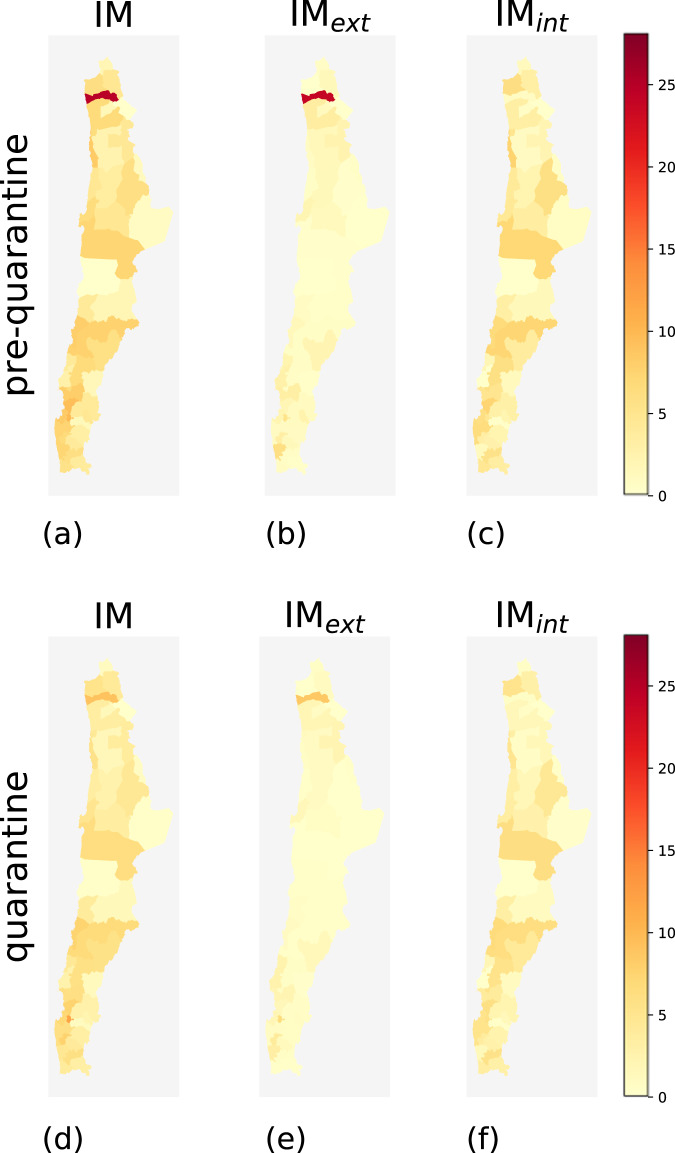
Choropleth maps of IM, IM_*int*_ and IM*ext* for the comunas in northern Chile for the pre-quarantine (first row) and the quarantine (second row) periods.

**Fig. 3:**
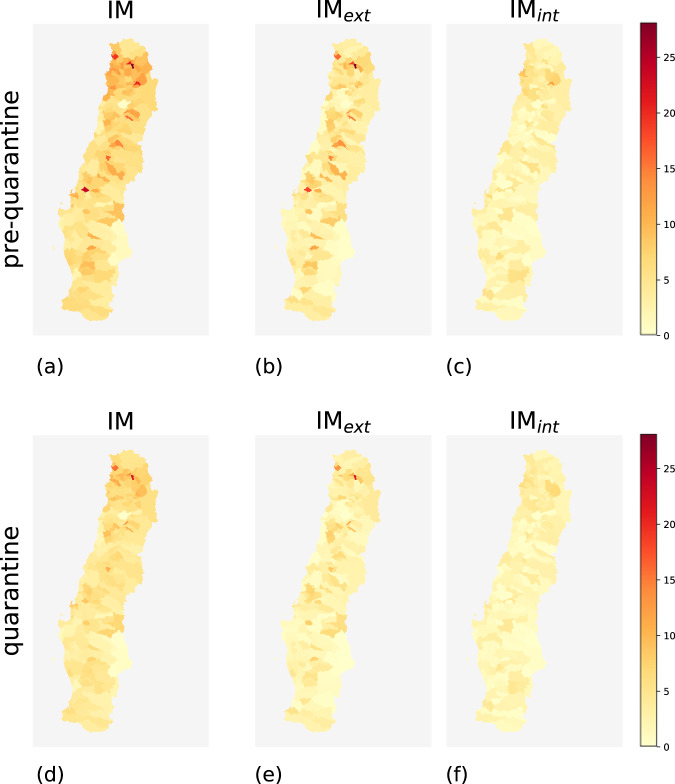
Choropleth maps of IM, IM*int* and IM_*ext*_ for the comunas in central Chile for the pre-quarantine (first row) and the quarantine (second row) periods.

**Fig. 4:**
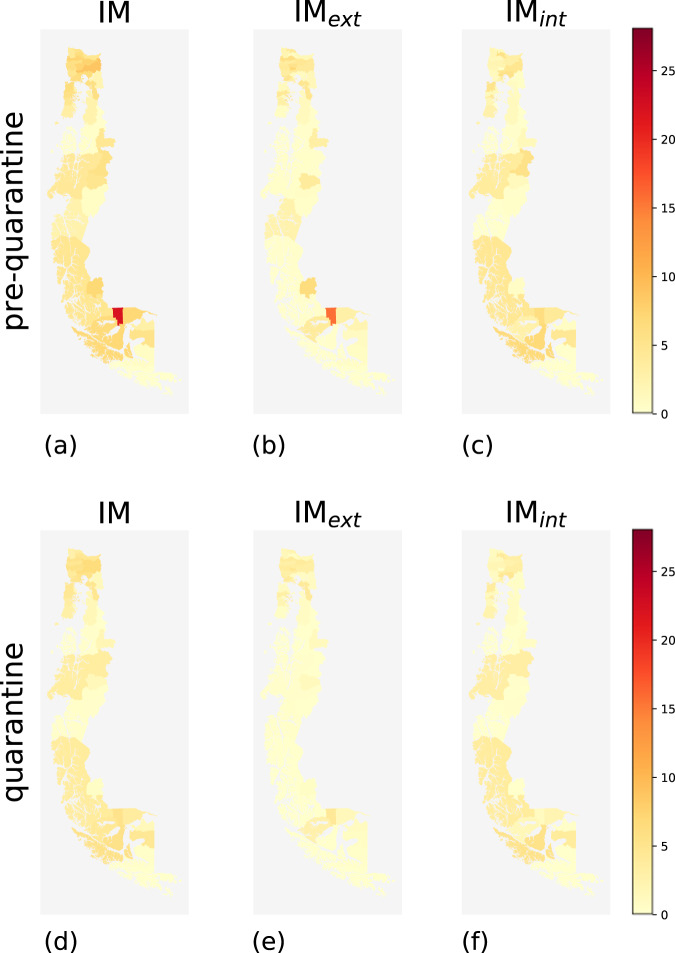
Choropleth maps of IM, IM_*int*_, and IM_*ext*_ for the comunas in southern Chile for the pre-quarantine (first row) and the quarantine (second row) periods.

**Fig. 5:**
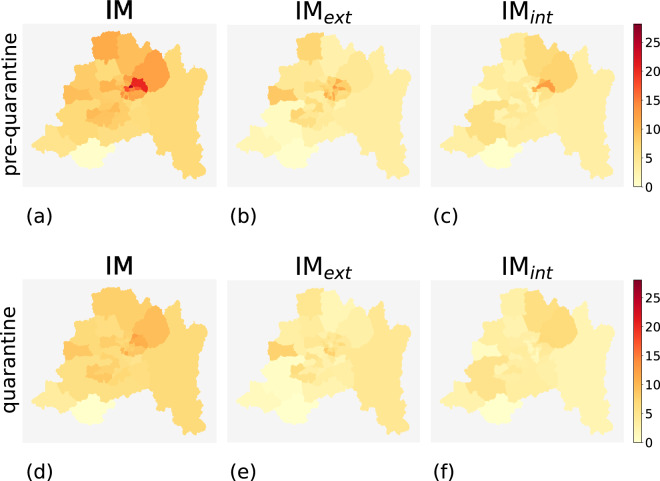
Choropleth maps of IM, IM_*int*_ and IM_*ext*_ for the comunas in the metropolitan area of Santiago de Chile for the pre-quarantine (first row) and the quarantine (second row) periods.

During the pre-quarantine period, comunas with high mobility indices and comunas with low mobility indices coexist. Geographically, high-mobility comunas are concentrated near urban areas such as the capital Santiago and, in general, in the center of the country (Figs. 2a, 3a, 4a, and 5a). The northern and southern parts of Chile have fewer high-mobility comunas. The comunas with the highest mobility registered during the pre-quarantine period are located in the regions of Metropolitana de Santiago, Arica y Parinacota, Valparaíso, Ñuble, and Magallanes (Table 3).

**Table 3:** Values of IM, IM_*ext*_ and *IM*_*int*_ of the ten comunas with the highest IM computed between March 9th and March 15th, 2020.

Pre-quarantine period					
	Comuna	Region	IM	*IM*_*ext*_	*IM*_*int*_
1	Rinconada	Valparaíso	30.37	27.96	2.42
2	Providencia	Metropolitana de Santiago	25.29	12.58	12.71
3	Camarones	Arica y Parinacota	24.62	23.77	0.85
4	Ranquil	Ñuble	23.87	18.33	5.54
5	Laguna Blanca	Magallanes	21.92	15.75	6.18
6	Panquehue	Valparaíso	20.93	19.02	1.90
7	Vitacura	Metropolitana de Santiago	20.40	10.54	9.86
8	Las Condes	Metropolitana de Santiago	20.22	7.79	12.42
9	Zapallar	Valparaíso	19.26	15.98	3.28
10	Santiago	Metropolitana de Santiago	17.44	6.97	10.48

As an example, Rinconada (Valparaíso region) has IM = 30.37, meaning that the number of movements within, to, or from that comuna is around 30 times higher than the estimated number of users that reside in Rinconada.

**Fig. 6:**
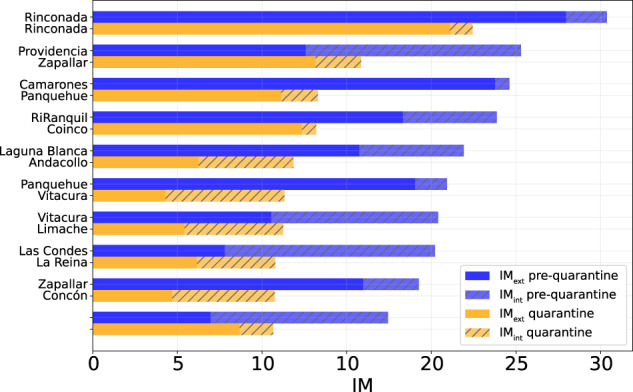
Values of IM, IM_*int*_, and IM_*ext*_ of the comunas in the top 10 ranking computed for the pre-quarantine and quarantine period. The coupled bars represent comunas corresponding to the same position in the rank.

**Fig. 7:**
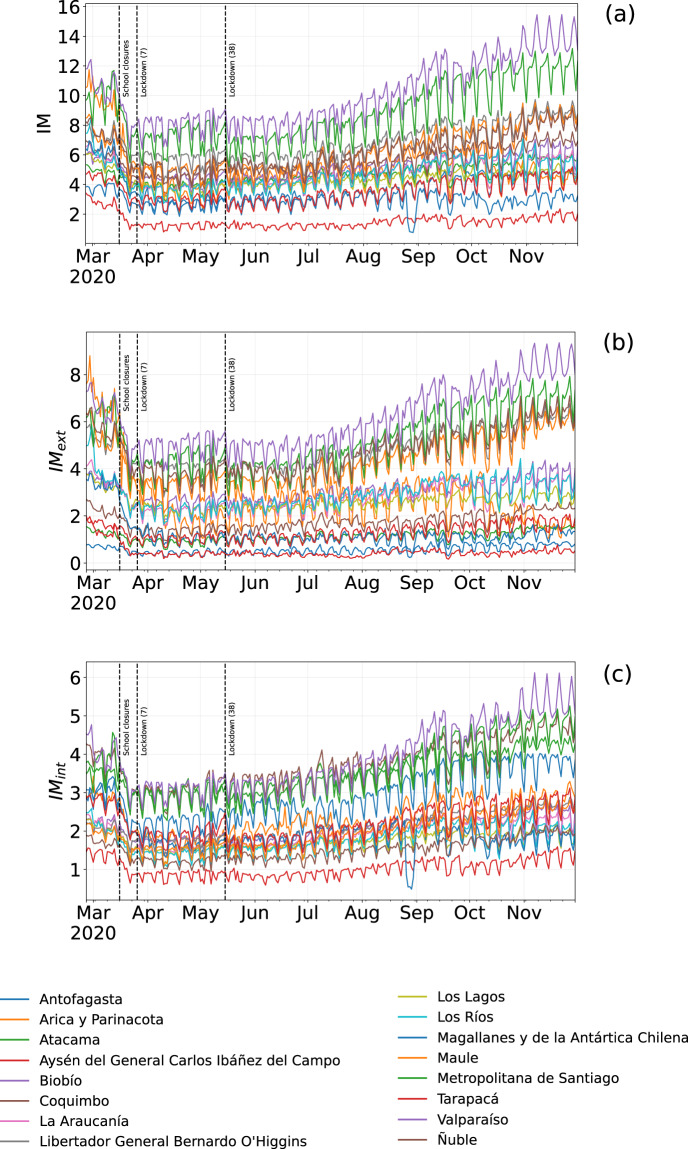
Evolution of IM (**a**), *IM*_*ext*_ (**b**) and *IM*_*int*_ (**c**) from March to September 2020 for the 16 regions in Chile. The curves are sorted in descending order respect to the relative index of mobility of the corresponding comuna. The vertical lines denote important dates regarding NPIs in Chile; the number in parentheses indicates the number of comunas subject to that restriction.

The top-ten comunas with the highest mobility indices change during the quarantine period, except for Rinconada in the region of Valparaíso (Table 4), mirroring the different degree of reduction in human mobility in the Chilean regions (Fig. 6). All regions show a reduction in all three mobility indices during the quarantine period, albeit with different intensities (Fig. 7). At the comuna level, high-mobility comunas are rare and clustered near the large urban areas located in central Chile (Figs. 2–5).

**Table 4:** Values of IM, IM_*ext*_ and IM_*int*_ of the ten comunas with the highest IM computed over the period from June 22nd and June 28th, 2020.

Quarantine period					
	Comuna	Region	IM	IM_*ext*_	IM_*int*_
1	Rinconada	Valparaíso	22.44	21.09	1.35
2	Zapallar	Valparaíso	15.84	13.16	2.68
3	Panquehue	Valparaíso	13.30	11.13	2.17
4	Coinco	Libertador Gen. B. O’Higgins	13.20	12.36	0.84
5	Andacollo	Coquimbo	11.85	6.25	5.60
6	Vitacura	Metropolitana de Santiago	11.33	4.29	7.04
7	Limache	Valparaíso	11.25	5.41	5.84
8	La Reina	Metropolitana de Santiago	10.78	6.16	4.62
9	Concón	Valparaíso	10.75	4.69	6.06
10	Villa Alegre	Maule	10.67	8.67	1.99

As an example, Rinconada (Valparaíso region) has IM = 22.44, meaning that the number of movements within, to, or from that comuna is around 22 times higher than the estimated number of users that reside in Rinconada.

**Fig. 8:**
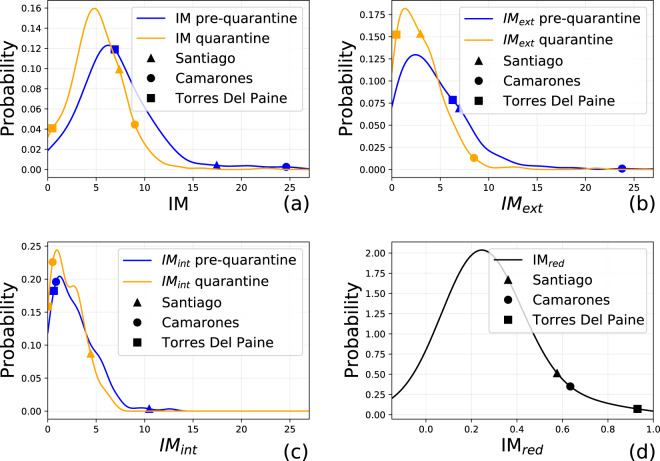
Distributions of IM (**a**), *IM*_*ext*_ (**b**) and *IM*_*int*_ (**c**) for the pre-quarantine (blue) and quarantine (orange) periods, with the average values of three comunas: Santiago, Camarones and Torres Del Paine. (d) Distribution of *IM*_*red*_ for all the Chileans comunas.

These results are supported by the distributions of the mobility indices of the two periods (Fig. 8). There is a clear shift towards the left of the distribution of the IM index (Fig. 8a): *(i)* the average IM during the quarantine period (5.16 ± 2.74) is 27.6% lower than the average IM during the pre-quarantine period (7.13 ± 4.15); *(ii)* the distribution of IM during the quarantine period is more skewed to the left, showing a decrease of the mobility in Chile during the selected days. Regarding IM_*int*_ and IM_*ext*_, we observe no net shift of the curve, but rather a flattening, suggesting that intra- and inter-comuna movements decreased during the quarantine (Fig. 8b,c).

**Fig. 9:**
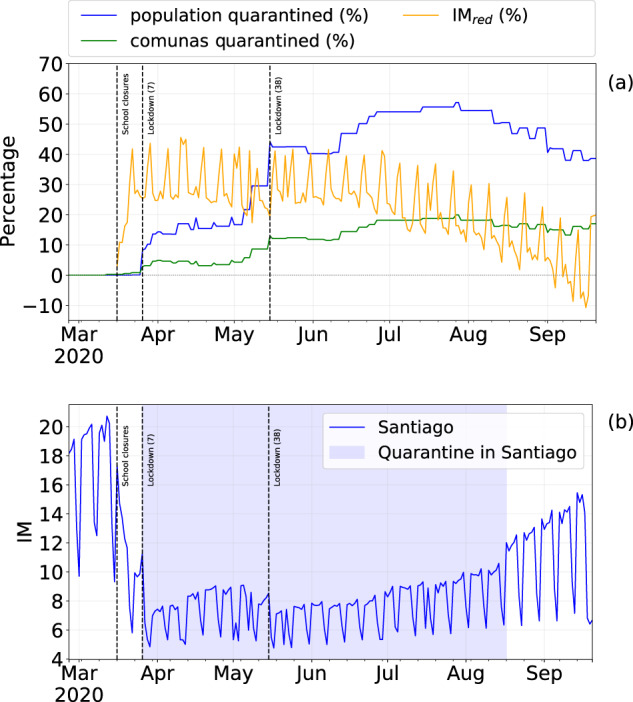
(**a**) Percentage of population under quarantine and the percentage of mobility reduction IM_*red*_ from February 26th to September 20th, 2020. (**b**) Evolution of IM index in Santiago; the blue area denotes the quarantine period. The vertical lines denote important dates regarding NPIs in Chile; the number in parentheses indicates the number of comunas subject to that restriction.

We further analyze the reduction of the mobility defining *IM*_*red*_ as the relative reduction of the *IM* index in the quarantine period with respect to the pre-quarantine period. The distribution of *IM*_*red*_ shows that a large number of comunas have a reduced mobility, following Chilean government interventions, by an average of 25.37% ± 43.2 (Fig. 8d). However, comunas that were not in quarantine during the quarantine period do not reduce their mobility significantly (Fig. 9a).

The percentage of population that live in comunas where the authorities applied NPIs increases with time (Fig. 9a) reaches its peak (≈57%) in late July 2020. With the increase of the number of people under quarantine, *IM*_*red*_ initially increases, but it slightly decreases over time even if both the number of individuals and the number of comunas under quarantine increase. This phenomenon suggests that mobility restrictions are more effective in the short-medium term and become less effective as time goes by, and it can be observed both at regional (Fig. 7) and comuna level (Fig. 9a,b).

Unfortunately, we do not have ground truth data to compare our data with because there are no official indices at the comuna level in Chile. However, other mobility reports do exist for the same area and period at the regional level (not comunas), such as Google Mobility Reports^13^. By aggregating our data at the regional level and comparing them with Google’s data, we find a strong Pearson correlation (*r* = 0.7), suggesting that our mobility index is reflecting mobility trends captured by other reliable data sources.

### Limitations of our dataset

Mobile phone records are sparse and irregular in time, leading to gaps between the user’s actual trajectory and the trajectory that can be inferred from their digital trace^15^. Chen *et al*.^60^ propose an algorithm to reconstruct individual trajectories from CDRs by recovering the unspecified positions of each user. They revisit the seminal work of Gonzalez *et al*.^23^, in which the authors show that heavy-tails characterise the distributions of (charateristic) distances traveled by individuals, showing that CDRs preserve the mobility patterns observed in the reconstructed (denser) trajectories, though slightly underestimating long trips and overestimating short ones^60^. Considering that in our study we use XDRs, which are way denser than CDRs, we can assume that the mobility traces of individuals represented in our dataset do not differ significantly from the actual user’s trajectory.

## Data Availability

The up-to-date data are available from the general repository of the Ministry of Science of Chile at: https://raw.githubusercontent.com/MinCiencia/Datos-COVID19/master/output/producto33/IndiceDeMovilidad.csv (IM indeces), and https://github.com/MinCiencia/Datos-COVID19/blob/master/output/producto29/Cuarentenas-Activas.csv (quarantines). The code to download the up-to-date data automatically and to reproduce the analysis in our paper is available at^58^.
